# High Glucose Predisposes Gene Expression and ERK Phosphorylation to Apoptosis and Impaired Glucose-Stimulated Insulin Secretion via the Cytoskeleton

**DOI:** 10.1371/journal.pone.0044988

**Published:** 2012-09-14

**Authors:** Ronne Wee Yeh Yeo, Kaiyuan Yang, GuoDong Li, Sai Kiang Lim

**Affiliations:** 1 Institute of Medical Biology, Agency for Science, Technology and Research (A*STAR), Singapore, Singapore; 2 Graduate School for Integrative Sciences and Engineering, National University of Singapore, Singapore, Singapore; 3 Raffles Institution, Singapore, Singapore; 4 Department of Clinical Research, Singapore General Hospital, Singapore, Singapore; 5 Department of Surgery, Yong Loo Lin School of Medicine, National University of Singapore, Singapore, Singapore; University of Bremen, Germany

## Abstract

Chronic high glucose (HG) inflicts glucotoxicity on vulnerable cell types such as pancreatic β cells and contributes to insulin resistance and impaired insulin secretion in diabetic patients. To identify HG-induced cellular aberrations that are candidate mediators of glucotoxicity in pancreatic β cells, we analyzed gene expression in ERoSHK6, a mouse insulin-secreting cell line after chronic HG exposure (six-day exposure to 33.3 mM glucose). Chronic HG exposure which reduced glucose-stimulated insulin secretion (GSIS) increased transcript levels of 185 genes that clustered primarily in 5 processes namely cellular growth and proliferation; cell death; cellular assembly and organization; cell morphology; and cell-to-cell signaling and interaction. The former two were validated by increased apoptosis of ERoSHK6 cells after chronic HG exposure and reaffirmed the vulnerability of β cells to glucotoxicity. The three remaining processes were partially substantiated by changes in cellular morphology and structure, and instigated an investigation of the cytoskeleton and cell-cell adhesion. These studies revealed a depolymerized actin cytoskeleton that lacked actin stress fibers anchored at vinculin-containing focal adhesion sites as well as loss of E-cadherin-mediated cell-cell adherence after exposure to chronic HG, and were concomitant with constitutive ERK1/2 phosphorylation that was refractory to serum and glucose deprivation. Although inhibition of ERK phosphorylation by PD98059 promoted actin polymerization, it increased apoptosis and GSIS impairment. These findings suggest that ERK phosphorylation is a proximate regulator of cellular processes targeted by chronic HG-induced gene expression and that dynamic actin polymerization and depolymerization is important in β cell survival and function. Therefore, chronic HG alters gene expression and signal transduction to predispose the cytoskeleton towards apoptosis and GSIS impairment.

## Introduction

Diabetes mellitus is an epidemic metabolic disease with mild early symptomatic manifestations that as a result of poor disease management, often lead to severe complications such as cardiovascular disease, limb amputation, diabetic retinopathy, renal failure, diabetic neuropathy (http://www.who.int/topics/diabetes_mellitus/en/; accessed August 2011). Type 2 (or non-insulin-dependent or adult-onset) diabetes (T2D) which is the more prevalent form, is caused by reduced insulin release, peripheral insulin resistance, and insulin failure to suppress glucose production [1]. The pathophysiology of diabetic complications is a complex multifactorial process that ensues primarily from hyperglycemia, and targets vulnerable cells such as capillary endothelial cells in the retina, mesangial cells in the renal glomerulus, neurons and Schwann cells in peripheral nerves [2], and pancreatic β cells. Clinical studies have demonstrated that hyperglycemia toxicity causes deterioration of pancreatic β cell mass and function leading to the progression of diabetes and development of diabetic complications in diabetic patients [3], [4], [5] [reviewed by Wajchenberg [6]]. The vulnerability of pancreatic β cells and other diabetes-associated cell types to hyperglycemia-induced tissue toxicity (or glucotoxicity) has been attributed to their inability to reduce glucose uptake when exposed to hyperglycemia [2]. Excessive glucose uptake has been shown to induce biochemical changes such as increased flux through the polyol pathway, enhanced production of advanced glycation end products (AGEs), activation of PKC, and increased activity of the hexosoamine pathway [2]. These biochemical changes have the potential to increase reactive oxygen species production and to modify proteins and lipids that could result in global cellular damage and severe phenotypic alterations such as mitochondria dysfunction [7], endoplasmic reticulum stress [8], increased intracellular calcium and cell death [9]. However, the progression from these glucotoxicity-induced cellular damages to deterioration or loss of specific β cell functions such as loss of glucose sensitivity and impairment of glucose-stimulated insulin secretion (GSIS), [10], [11], [12], [13], [14] and ultimately β cell apoptosis [15], [16], [17], remains poorly characterized. Since β cell dysfunction has been shown to be central to the development and progression of T2D [18], timely interventional therapies to preserve the important β cell function of GSIS and viability are likely to improve the prognosis of the disease [19], [20].

In view of the multiple reported effects of hyperglycemia on β cells, we attempted to expand the current understanding through gene expression profiling to identify cellular changes that are induced by exposure to high glucose. We employed mouse ESC-derived insulin-producing ERoSHK6 cells as a surrogate β cell model for this study. This clonal and highly scalable cell line displays the defining functional properties of bona fide pancreatic β cells. ERoSHK6 cells synthesize and store insulin in typical intracellular vesicles. Under stimulation by secretagogues such as glucose, tolbutamide and glibenclamide, these cells close their ATP-sensitive K^+^ channels, leading to membrane depolarization, opening of Ca^2+^ channels and the subsequent release of insulin and C-peptide in equimolar ratio, a mechanism resembling that of primary β cells. Most importantly, these cells can reverse hyperglycemia when grafted into streptozotocin-treated mice [21]. ERoSHK6 cells also exhibit biochemical pathways that are highly characteristic of β cells, such as the pentose phosphate pathway, clathrin-mediated endocytosis and peroxisome proliferator-activated receptor (PPAR) signaling pathway [22]. ERoSHK6 cells are ideal for investigating the effects of chronic hyperglycemia on pancreatic β cells not only for their close resemblance to β cells but also for their capacity to be propagated in culture for extended periods, unlike primary β cells.

To assess the effect of chronic exposure to high glucose on gene expression, ERoSHK6 cells were exposed to HG (33.3 mM) for six days. This exposure reduced GSIS and analysis of the cellular RNA by hybridization of a microarray of gene probes identified 185 genes that are highly upregulated in HG-treated ERoSHK cells. These genes were involved primarily in regulating cell structure and survival. In concordance with our microarray analysis, we observed that high glucose led to increased apoptosis and changes in ERoSHK colony morphology, depolymerisation of actin and loss of E-cadherin-mediated cell-cell contact. Together, these observations implicated the cytoskeleton as a major target of HG-mediated cellular aberrations.

The critical role of the cytoskeleton in stimulated insulin secretion by pancreatic β cells has long been recognized [23], [24], [25]. Insulin exocytosis has been observed to occur via at least three different modes of granule fusion with the plasma membrane. One mode is fusion with pre-docked granules, the second is the immediate fusion of newly recruited granules to the plasma membrane and the third mode is fusion with newly recruited and docked granules [26]. Insulin exocytosis has also been shown to occur via partial fusion with the plasma membrane (termed “kiss-and-run exocytosis”) [27], [28]. Irrespective of the mode of granule fusion with the plasma membrane, the physical execution of GSIS in pancreatic β cells requires dynamic and transient remodeling of the cytoskeleton to move secretory vesicles to the inner surface of the plasma membrane, dock the granules and exocytose the contents of the granules by membrane fusion and fission [29], [30] It has been observed that depolymerization and polymerization of F-actin are necessary to execute GSIS. For example, F-actin depolymerisation has been shown to enhance GSIS in MIN6 β cells [31] while inhibition of RhoGDI which activates Cdc42 to promote actin polymerization led to increased GSIS [32], [33]. These diametrical observations could be reconciled by the need to alternate between polymerized and depolymerized F-actin cytoskeleton to move, dock and fuse secretory vesicles for GSIS, and a predilection for either a polymerized or depolymerized state would compromise GSIS. Incidentally, an alternating actin polymerization and depolymerization has been shown to be important in cellular survival [reviewed in [34]]. This need to maintain a stimuli-responsive cytoskeleton connotes an exquisitely regulated cytoskeleton in pancreatic β cells. Indeed, many cytoskeletal components e.g. focal adhesions [35], F-actin [24], [36], [37], [38], [39], [40], and regulatory small G proteins e.g. Cdc42 and Rac1 [41], [42], [43] have been implicated in the regulation of insulin secretion.

This study also implicated ERK signaling in HG-induced changes in ERoSHK colony morphology, depolymerisation of actin and the loss of E-cadherin-mediated cell-cell contact. HG-induced ERK activation was found to be sustained and refractory to serum starvation, and therefore not likely to be compatible with the dynamic actin polymerization and depolymerization necessary for the execution of GSIS and cellular survival. ERK1/2 phosphorylation which is downstream of MAPK signaling has been associated with actin cytoskeleton remodeling, focal adhesion remodeling and GSIS [31], [44]. The role of ERK1/2 in β cell function has been implicated in diametrical processes and remains to be clarified. For example, glucose-induced ERK1/2 phosphorylation has been implicated in the long term deleterious effects of high glucose on β cell, namely apoptosis and impaired insulin secretory function [45] while adiponectin- or insulin-induced ERK1/2 phosphorylation improves cell survival and insulin secretory function [46], [47].

## Results

### High glucose impaired insulin secretion in ERoSHK6 cells

**Figure 1:**
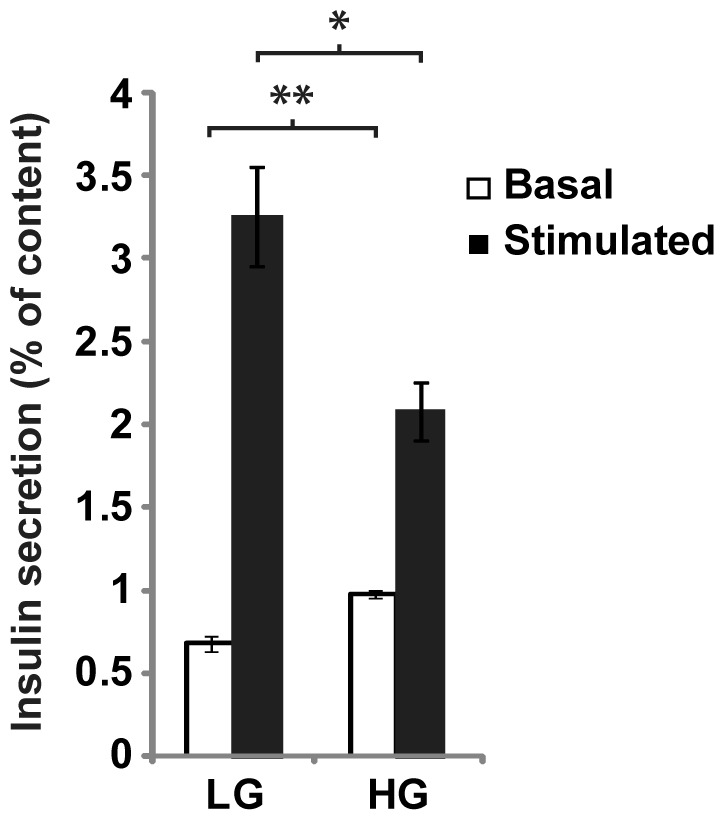
High glucose impaired insulin secretion in ERoSHK. Insulin secretion of ERoSHK_LG_ and ERoSHK_HG_ in response to 2.8 mM (basal) and 16.7 mM (stimulated) glucose, expressed as a percentage of total insulin content. Data are presented as mean ± s.d.; n = 3. *, *P*<0.005; **, *P*<0.001.

To determine if chronic high glucose was detrimental to ERoSHK6 cells, the cells were cultured in INS medium containing either LG or HG. Cells exposed to 6 days of LG or HG treatment will hereafter be referred to as ERoSHK_LG_ or ERoSHK_HG_ respectively. Since HG is known to cause β cell dysfunction, we assessed insulin secretion by ERoSHK_LG_ and ERoSHK_HG_. Relative to ERoSHK_LG_, basal insulin secretion of ERoSHK_HG_ when assayed in the presence of 2.8 mM glucose was increased by 43.70% (*P*<0.001) while its glucose stimulated insulin secretion (GSIS) as assayed in the presence of 16.7 mM glucose was reduced by 36.33% in ERoSHK_HG_ (*P*<0.005) (Fig. 1).

### Changes in gene expression of ERoSHK_HG_ cells targeted cell survival and structure

**Figure 2:**
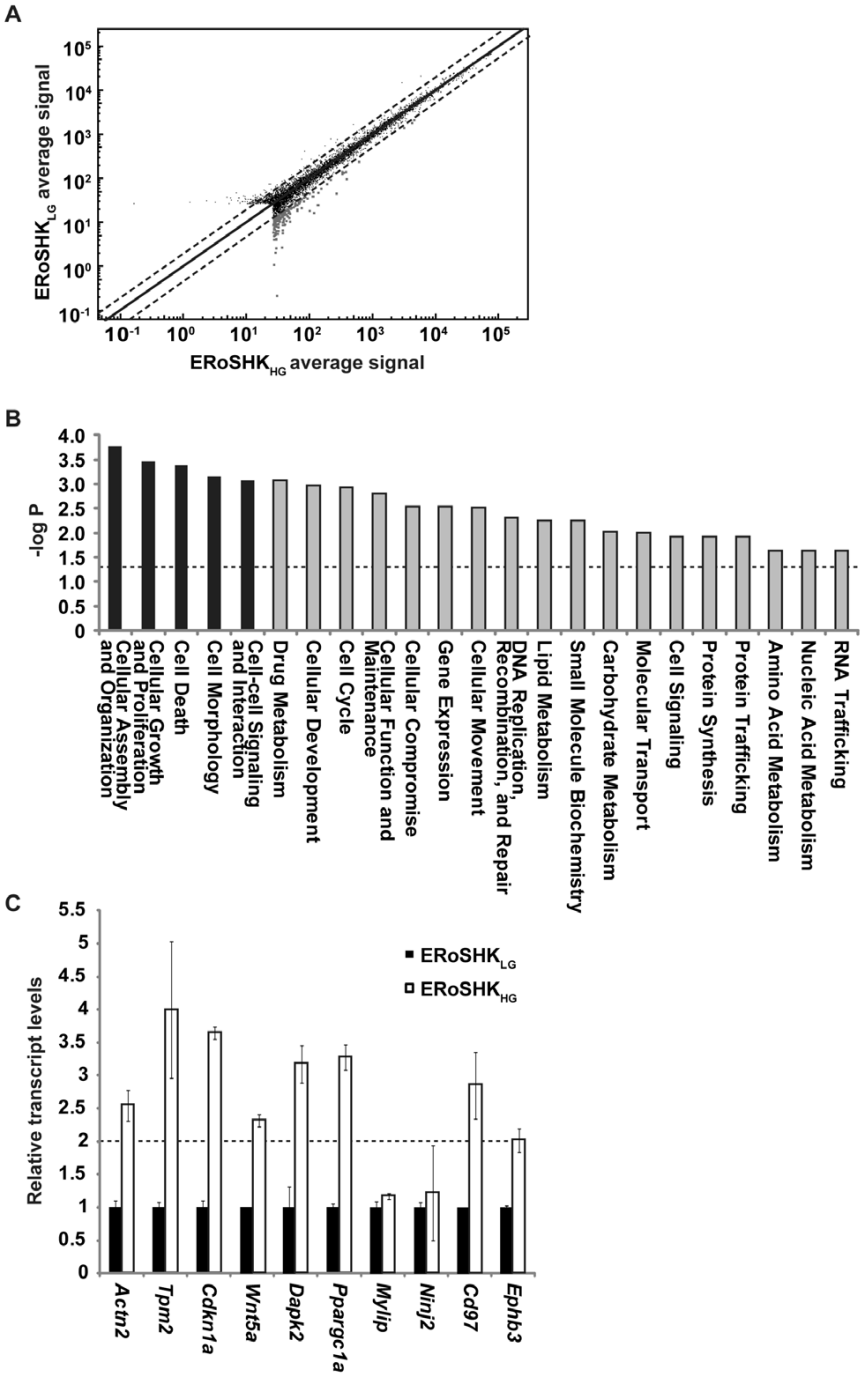
High glucose enhanced the expression of genes that regulate cell survival and structure in ERoSHK. **a** Scatter plot was created using Illumina BeadStudio, to display the 6813 genes expressed by both ERoSHK_LG_ and ERoSHK_HG_ and their average detection signal on logarithmic scales. Dashed lines represent 2.0-fold difference boundaries and grey dots represent the 185 genes up-regulated by ERoSHK_HG_. **b** The 185 up-regulated genes were uploaded onto Ingenuity Pathway Analysis, and over-represented in 23 molecular and cellular functions with *P*<0.05 (dashed line). The top 5 most significant functions are represented by black bars. **c** Expression levels of 10 randomly selected genes, 2 from each of the top 5 most significant functions, as determined by quantitative PCR. Transcript levels are first normalized to *Actb* endogenous control, and then to those of ERoSHK_LG_. Data are presented as mean ± s.d.; n = 3.

For a better understanding of hyperglycemia-induced cellular changes that result in β cell dysfunction and death, we studied the global gene expression profile of ERoSHK_LG_ and ERoSHK_HG_ by hybridizing labeled cRNA to Illumina BeadArray containing about 25,700 unique features. After filtering out genes with detection values below the confidence level of 95%, we detected the expression of 6628 genes in ERoSHK_LG_ versus 6680 genes in ERoSHK_HG_. Of these, 6495 genes were expressed at similar levels (<2.0-fold difference) between the two samples. ERoSHK_HG_ up-regulated the expression of 185 genes as compared to ERoSHK_LG_, based on a fold-change cut-off of ≥2.0 (Figure 2A, list of genes in Table S2). These 185 genes highly expressed in ERoSHK_HG_ were analyzed by Ingenuity Pathway Analysis (Ingenuity Systems), which clustered the genes based on their cellular functions. The 5 most significant cellular functions (*P*<0.05) are: (1) cellular assembly and organization; (2) cellular growth and proliferation [38]; (3) cell death; (4) cell morphology; and (5) cell-to-cell signaling and interaction (Fig. 2B). The genes that are over-represented in these cellular functions can be found in Table S3. To further validate these results from microarray analysis, the expression levels of a random sample of 10 genes (two from each of these five significant cellular processes) were evaluated by qPCR. The 10 genes were *Actn2* and *Tpm2* involved in cellular assembly and organization; *Cdkn1a* and *Wnt5a* involved in cellular growth and proliferation; *Dapk2* and *Ppargc1a* involved in cell death; *Mylip* and *Ninj2* involved in cell morphology; and *Cd97* and *Ephb3* involved in cell-to-cell signaling and interaction. Of the ten genes, 8 genes were indeed upregulated by more than 2.0-fold in ERoSHK_HG_ as compared to ERoSHK_LG_, in concordance with the microarray analysis (Fig. 2C).

### HG inhibited population growth by increased apoptosis with no significant effect on rate of cell division

**Figure 3:**
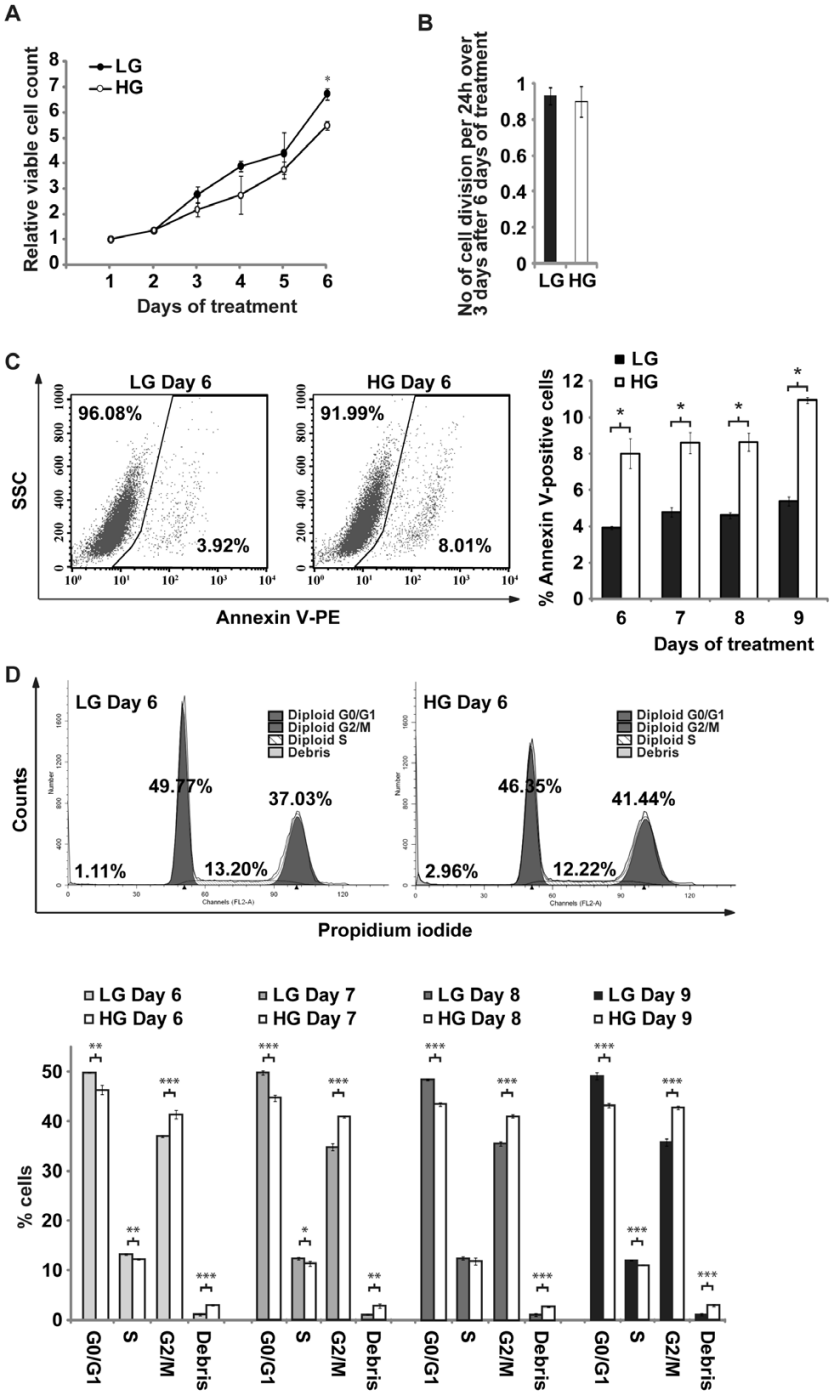
HG inhibited population growth by increased apoptosis with no significant effect on rate of cell division. **a** Cells were cultured in LG or HG conditions and total number of viable cells was quantified each day as determined by Guava Viacount assay. Data are normalized to the number of cells seeded on day 0, and represented as mean ± s.d.; n = 3. *, *P*<0.001. **b** ERoSHK cells pretreated with LG and HG for 6 days were stained with CFSE and the rate of loss of fluorescence signal was monitored over 72 h by flow cytometry. The number of divisions per 24 h during the 72 h period after 6 days of LG and HG treatment is graphically represented (mean ± s.d.; n = 3). **c** *Left panel*, ERoSHK_LG_ and ERoSHK_HG_ were stained with phycoerythrin-conjugated Annexin V and dye fluorescence was measured by flow cytometry. The gates on scatter plots of side-scatter (SSC) versus phycoerythrin fluorescence intensity represent Annexin V-positive cells. *Right panel*, percentages of Annexin V-positive cells were assessed daily over a period of 72 h and graphically represented. Data are presented as mean ± s.d.; n = 3. *, *P*<0.001. **d** *Upper panel*, ERoSHK_LG_ and ERoSHK_HG_ were fixed, stained with propidium iodide (PI) and dye fluorescence was measured by flow cytometry. Histogram plot of cell number versus PI fluorescence intensity was deconvoluted using ModFit LT software. Percentage of cells in each phase of the cell cycle is depicted above their respective peaks. *Lower panel*, the experiment was further repeated 24, 48 and 72 h post treatment and percentages of cells in the different cell cycle phases are graphically represented. Data are presented as mean ± s.d.; n = 3. *, *P*<0.05; **, *P*<0.005; ***, *P*<0.001.

Cellular growth and proliferation, and cell death were two of five most significant cellular functions that were computationally predicted to be modulated by the HG-induced changes in gene expression. Consistent with these predictions, viable cell population for ERoSHK_LG_ and ERoSHK_HG_ increased 6.7- and 5.5-fold respectively (*P*<0.001) (Fig. 3A). The first significant difference occurred at day 6. To ascertain if this difference was due to reduced cell division or increased apoptosis, the rate of cell division in ERoSHK_LG_ and ERoSHK_HG_ at day 6 and onwards was determined by labeling cells with CFSE and monitoring cellular fluorescence over 72 hours by flow cytometry. Assuming that cellular fluorescence was halved at each cell division, the number of cell division over time could be calculated as a function of loss in cellular fluorescence as described in Materials and Methods. The mean number of cell divisions per 24 h over a period of 72 h post-treatment for ERoSHK_LG_ and ERoSHK_HG_ were not significantly different at 0.93 versus 0.90 (*P*>0.05) (Fig. 3B), while the average duration of each cell division was 26.12 h versus 25.52 h respectively (Fig. S1). Therefore the reduced population growth in ERoSHK_HG_ cells was not due to reduced cell division but possibly increased cell death. Annexin V staining of ERoSHK_LG_ and ERoSHK_HG_ cells revealed that apoptosis was consistently higher in ERoSHK_HG_ (Fig. 3C). Although this increase was small, it was highly significant (*P*<0.001). This was further corroborated by cellular DNA content analysis. Relative to ERoSHK_LG_, ERoSHK_HG_ was arrested in G2/M arrest with less cells in G0/G1 phase (49.77% to 46.35%, *P*<0.005) and S phase (13.20% to 12.22%, *P*<0.005) but more in G2/M phase (37.03% to 41.44%, *P*<0.001). Consistent with the increased Annexin V-positive ERoSHK_HG_ cells, there was also more cellular debris in ERoSHK_HG_ culture as represented by the sub-G0 fraction (1.11% to 2.96%, *P*<0.001) (Fig. 3D, upper panel). These differences were maintained over the following 3 days (Fig. 3D, lower panel). Together, these observations indicated that HG reduced population growth of ERoSHK6 cells by inducing G2/M arrest and increasing apoptosis, without inhibiting the rate of cell division.

### ERoSHK_HG_ displayed altered cell morphology, disorganized actin cytoskeleton and reduced cell-cell adhesion

The computational prediction that the 185 genes highly expressed in ERoSHK_HG_ were involved in cellular assembly and organization, cell morphology and cell-to-cell signaling and interaction suggested that ERoSHK_HG_ would be morphologically different from ERoSHK_HG_. Further, as execution of these functions would all require modulation of the cytoskeleton and cellular adhesion, we speculated that the cytoskeleton and cellular adhesion structural changes in ERoSHK_HG_ were also modulated by chronic HG treatment.

**Figure 4:**
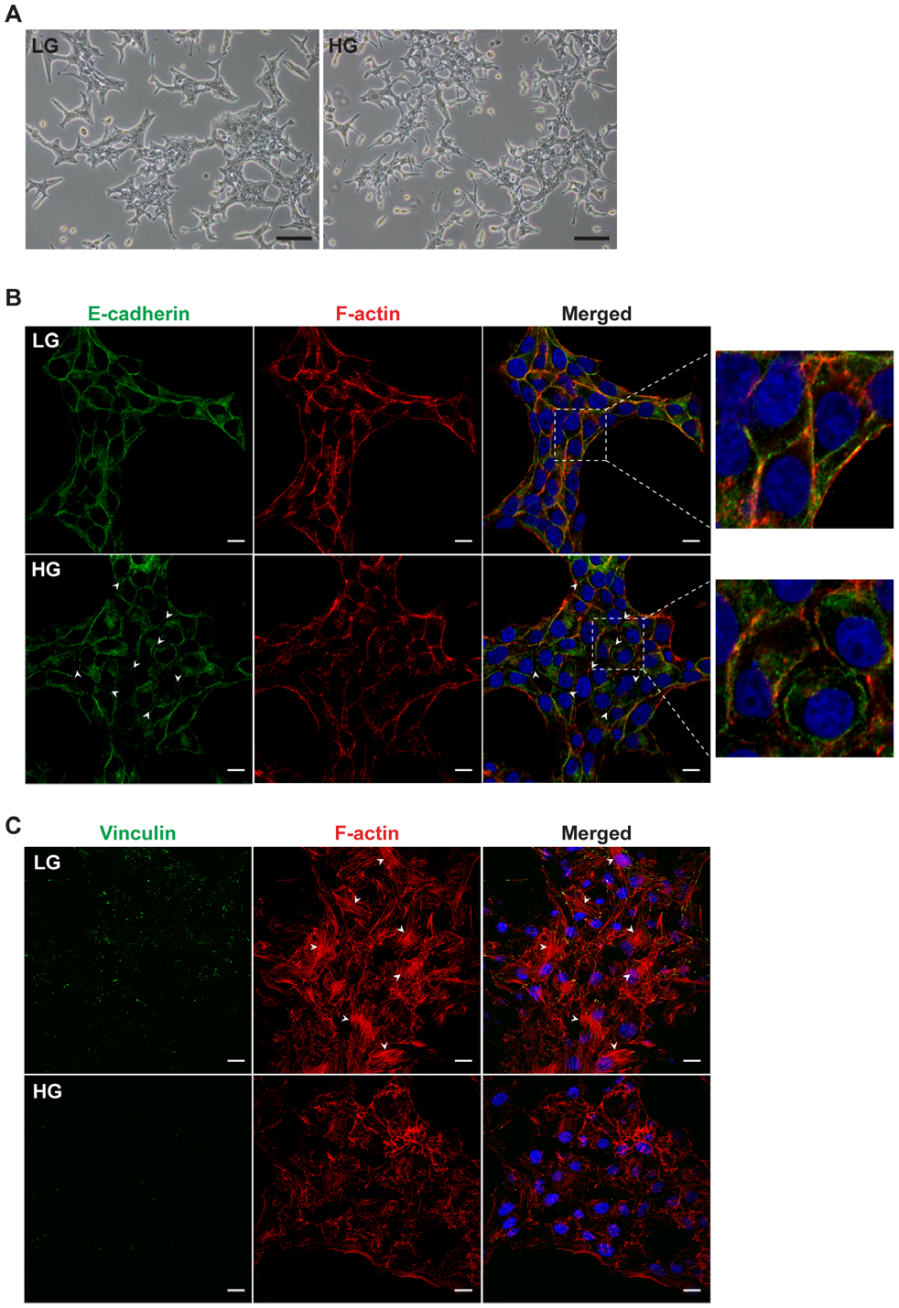
High glucose altered cell morphology, disrupted actin organization and reduced cell-cell adhesion in ERoSHK. **a** Representative phase contrast images of ERoSHK_LG_ and ERoSHK_HG_ showing changes in colony morphology. Scale bars, 100 µm. **b** Immunofluorescence staining for E-cadherin (green) in ERoSHK_LG_ and ERoSHK_HG_. Cells were counterstained with phalloidin (red) for F-actin and DAPI (blue) for nuclei. White arrowheads indicate sites of membrane separation. Parts of the merged image (box with white dashed lines) are magnified in the right panels to clearly visualize the co-localization of E-cadherin and cortical actin along the cell periphery, as well as gaps between adjacent cells. Scale bars, 10 µm. **c** Immunofluorescence staining for vinculin (green) in ERoSHK_LG_ and ERoSHK_HG_. Cells were counterstained with phalloidin (red) for F-actin and DAPI (blue) for nuclei. White arrowheads indicate actin stress fibers at the basal membrane. Scale bars, 10 µm.

Consistent with the computational prediction and its attendant implications, ERoSHK_HG_ colonies and cells were manifestly different from those of ERoSHK_LG_ which like MIN6 mouse pancreatic β cell line [48] grew in tightly packed colonies with close cell-cell contact. In contrast, ERoSHK_HG_ formed smaller colonies of loosely packed cells with reduced cell-to-cell contact (Fig. 4A). To assess the role of cytoskeletal organization and adhesion molecules in mediating this altered cellular morphology, both ERoSHK_LG_ and ERoSHK_HG_ were stained with fluorescence-tagged phalloidin and antibodies against E-cadherin. Consistent with the tightly packed cells in ERoSHK_LG_ colonies, adjacent cells within each colony shared common boundaries demarcated by E-cadherin (Fig. 4B). Staining with fluorescence-tagged phalloidin and antibodies against vinculin further revealed that the basement actin cytoskeleton in many of the ERoSHK_LG_ cells had F-actin stress fibers anchored at focal adhesion sites as indicated by vinculin. In contrast, many ERoSHK_HG_ cells within a colony did not share a common boundary with their neighbours and were physically separated by distinctly visible gaps between the E-cadherin demarcated membranes, indicating reduced cell-cell adhesion. Many of the ERoSHK_HG_ also did not display the focal adhesion-anchored F-actin stress fibers (Fig. 4C).

### Sustained high levels of ERK signaling in ERoSHK_HG_

**Figure 5:**
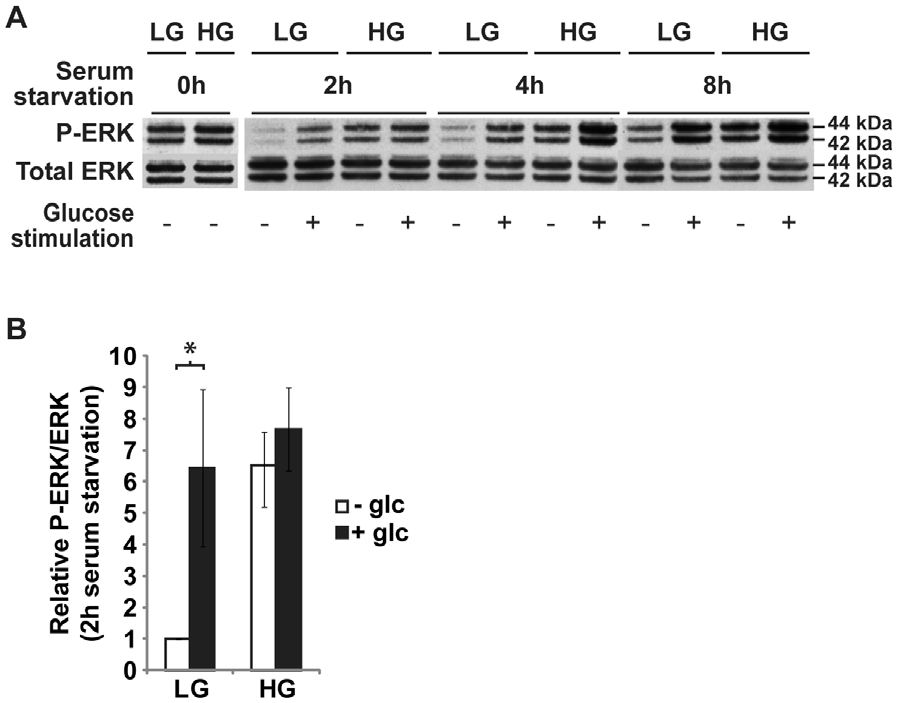
High glucose induced sustained ERK phosphorylation in ERoSHK. **a** *Left panel*, relative levels of phosphorylated ERK1/2 total ERK1/2 in ERoSHK_LG_ and ERoSHK_HG_ were analyzed by western blotting. *Right panel*, ERoSHK_LG_ and ERoSHK_HG_ were pre-incubated in KRBH buffer containing 0.1% BSA for 2, 4 and 8 h before being treated with or without a 10 mM glucose stimulation for 10 min. Relative levels of phosphorylated ERK1/2 and total ERK1/2 were then analyzed by western blotting. **b** 3 independent ERK phosphorylation assays were performed on ERoSHK_LG_ and ERoSHK_HG_ serum-starved for 2 h. The relative intensities of phosphorylated (P) and total ERK protein bands were quantified by densitometry and expressed as a ratio (P-ERK/ERK), normalized to that of unstimulated ERoSHK_LG_ cells. Data are presented as mean ± s.d.; n = 3. *, *P*<0.05.

Glucose is known to activate ERK1/2 in β cells during GSIS [49], [50], [51], and ERK1/2 activation has been implicated in actin cytoskeletal remodeling [31], [35], [52]. In addition, our gene expression analysis revealed that a factor reported to be involved in the activation of ERK, TNIP2 (ABIN-2) [53] was upregulated by HG. To determine if ERK signaling played a role in the cytoskeletal modifications during chronic high glucose exposure, pERK1/2 levels in ERoSHK_LG_ cells and ERoSHK_HG_ were assayed and were found to be comparable (Fig. 5A, left panel). However, we observed that 2, 4 and 8 hours of serum starvation, which expectedly abrogated ERK1/2 phosphorylation in ERoSHK_LG_ cells, failed to do so in ERoSHK_HG_ suggesting that ERK1/2 phosphorylation was refractory to downregulation by serum starvation and possibly to other regulatory controls, and ERoSHK_HG_ cells had constitutively active ERK signaling (Fig. 5A, right panel). As a consequence of the relatively high basal pERK1/2 level, glucose stimulation enhanced pERK1/2 level by 6.42±2.50-fold in ERoSHK_LG_ that were serum-starved for 2 hours (*P*<0.05). In serum-starved ERoSHK_HG_, the increase was not significant at 1.18±0.20-fold (*P*>0.05) (Fig. 5B).

### Abrogation of ERK signaling in ERoSHK_HG_ restored colony morphology, actin cytoskeletal disruption and loss of cell-cell contact

**Figure 6:**
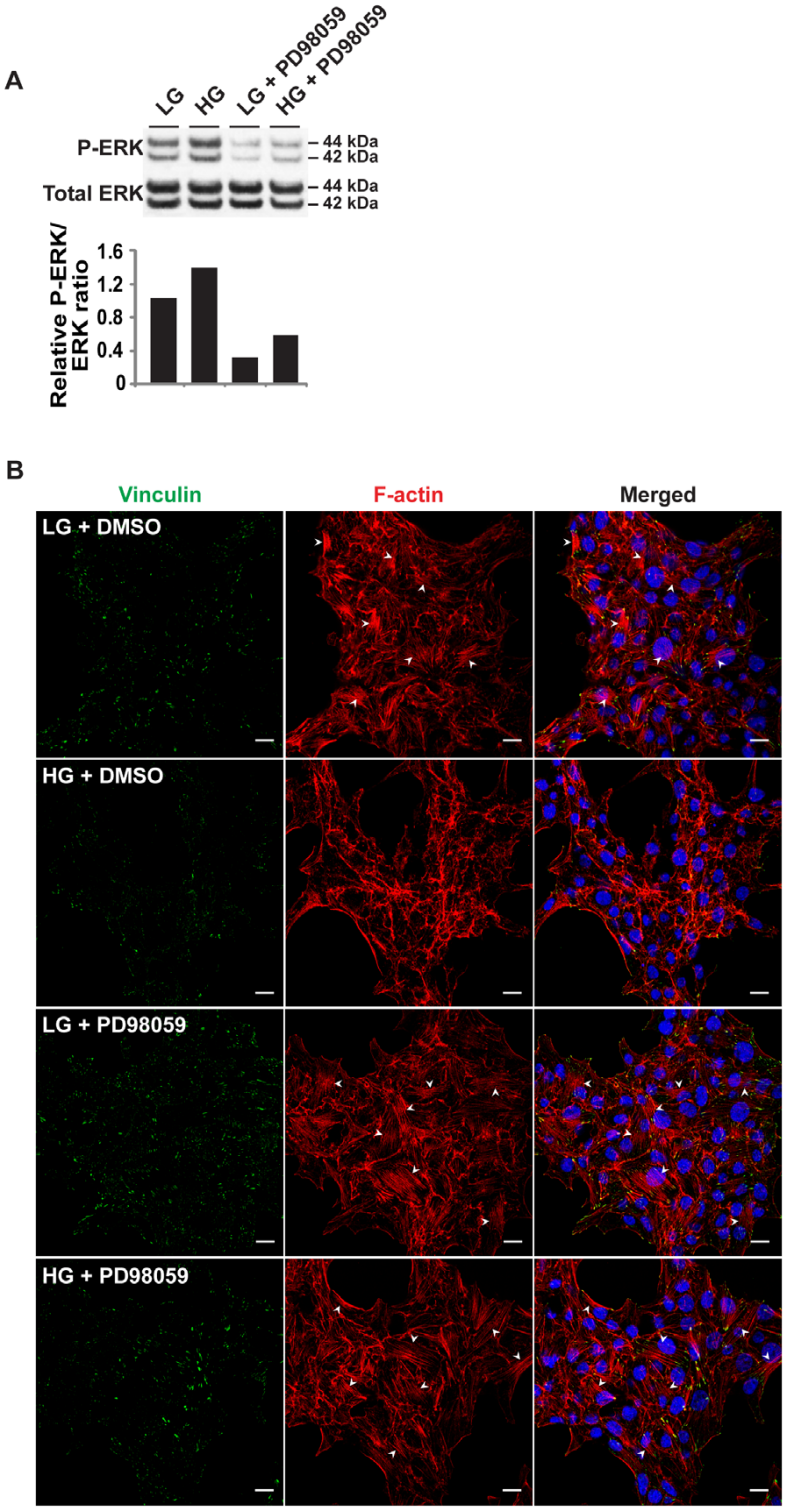
Inhibition of ERK phosphorylation in ERoSHK_HG_ restored actin cytoskeleton organization. **a** *Upper panel*, ERoSHK cells were cultured in LG or HG in the absence and presence of 25 µM of PD98059 for 6 days. Cells were lysed and relative amounts of phosphorylated ERK and total ERK were analyzed by western blotting. *Lower panel*, P-ERK/ERK ratios normalized to ERoSHK_LG_. Intensities of protein bands were quantified by densitometry. **b** Cells were fixed, immunostained for vinculin (green) and counterstained with phalloidin (red) for F-actin and DAPI (blue) for nuclei. White arrowheads indicate actin stress fibers at the basal membrane. Scale bars, 10 µm.

**Figure 7:**
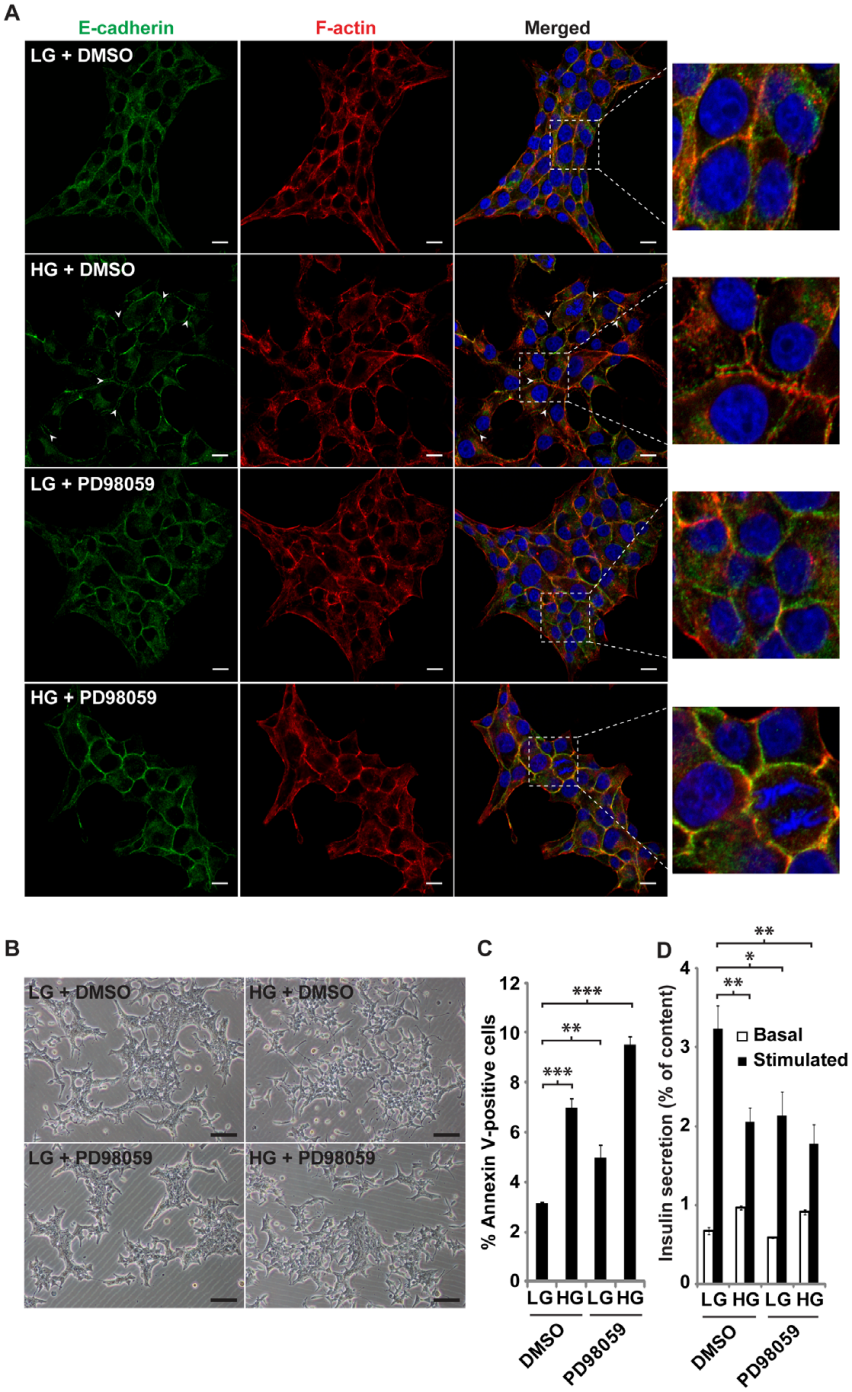
Inhibition of ERK phosphorylation in ERoSHK_HG_ restored cell-cell contact and colony morphology. **a** ERoSHK cells were cultured in LG or HG in the absence and presence of 25 µM of PD98059 for 6 days. Cells were fixed, immunostained for E-cadherin (green) and counterstained with phalloidin (red) for F-actin and DAPI (blue) for nuclei. White arrowheads indicate sites of membrane separation. Parts of the merged image (box with white dashed lines) are magnified in the right panels to clearly visualize the co-localization of E-cadherin and cortical actin along the cell periphery, as well as gaps between adjacent cells. Scale bars, 10 µm. **b** Representative phase contrast images of ERoSHK_LG_ and ERoSHK_HG_ treated with or without PD98059 to show changes in colony morphology. Scale bars, 100 µm. **c** ERoSHK_LG_ and ERoSHK_HG_ treated with or without PD98059 were stained with phycoerythrin-conjugated Annexin V and dye fluorescence was measured by flow cytometry. Percentages of Annexin V-positive cells are assessed and graphically represented. Data are presented as mean ± s.d.; n = 3. **d** Insulin secretion of ERoSHK_LG_ and ERoSHK_HG_ treated with or without PD98059 in response to 2.8 mM (basal) and 16.7 mM (stimulated) glucose, expressed as a percentage of total insulin content. Data are presented as mean ± s.d.; n = 3. *, *P*<0.05; **, *P*<0.005; ***, *P*<0.001.

The constitutively active pERK level in ERoSHK_HG_ and the documented role of pERK in the regulation of actin cytoskeleton [31], [35], [52] suggested that aberrant ERK signaling could be responsible for the disorganized actin cytoskeleton, loss of cell-cell adhesion and other changes in colony morphology of ERoSHK_HG_. To test this hypothesis, ERoSHK6 cells were cultured in LG or HG in the absence and presence of PD98059, a specific pharmacological inhibitor of MEK1/2 which phosphorylates ERK1/2 kinase [54]. To optimize the dose concentration of the inhibitor, we tested several inhibitor concentrations and determine that the lowest concentration required to reverse morphological changes in HG-treated cells was 25 µM. At this concentration, the inhibitor reduced ERK phosphorylation in both ERoSHK_LG_ and ERoSHK_HG_ from a similar pERK/ERK ratio (Fig. 5A) to the same level (Fig. 6A). The actin cytoskeleton of PD98059-treated ERoSHK_HG_ cells reverted to that of ERoSHK_LG_ cells with stress fibers anchored by focal adhesion sites while PD98059-treated ERoSHK_LG_ cells remained phenotypically similar to ERoSHK_LG_ (Fig. 6B). PD98059 also increased cell-cell contact within ERoSHK_HG_ colonies. PD98059-treated ERoSHK_HG_ cells were tightly packed within each colony and adjacent cells share common boundaries delineated by E-cadherin and cortical actin staining by rhodamine-phalloidin with no visible gaps between cells. Both PD98059-treated ERoSHK_LG_ and ERoSHK_HG_ resembled ERoSHK_LG_ (Fig. 7A). These cytoskeletal changes were also consistent with a change in the gross colony morphology of ERoSHK_HG_ to that of ERoSHK_LG_ (Fig. 7B). However, the rate of apoptosis in PD98059-treated ERoSHK_HG_ was not reduced to that in ERoSHK_LG_ or PD98059-treated ERoSHK_LG_ and remained high as in ERoSHK_HG_ (Fig. 7C). We observed that 5 µM PD98059 was sufficient to induce significant apoptosis in ERoSHK_HG_ (Fig. S2). GSIS was also attenuated in both PD98059-treated ERoSHK_LG_ and ERoSHK_HG_, at levels similar to ERoSHK_HG_ (Fig. 7D).

## Discussion

Metabolic overload of islets from chronic exposure to elevated level of nutrients (e.g. glucose) has been postulated to be important in the pathogenesis of T2D [55] and this importance is best evidenced by the effectiveness of meticulous glycemic control in slowing progression to diabetes and reducing the risk of microvascular and neurological complications of diabetes [56]. In pre-T2D patients, normoglycemia could be maintained as long as insulin resistance is adequately compensated by increased insulin secretion. When this β cell compensation is abrogated by β cell dysfunction and loss of β cell mass through apoptosis, T2D ensues with a fasting blood glucose level exceeding 5.5 mM. This ensuing hyperglycemia causes glucotoxicity and β cell phenotypic alterations to further exacerbate β cell dysfunction and increase apoptosis [18].

Over the years, multiple mechanistic models for the detrimental effects of hyperglycemia on the β cell have been proposed. In this study, we analysed global gene expression profiles of low and high glucose-treated ERoSHK6 cells as an unbiased approach to identify high glucose-induced cellular aberrations that are candidate mediators of glucotoxicity in pancreatic β cells.

ERoSHK6 is a clonal mouse embryonic stem cell-derived line that exhibits the salient functional features of pancreatic β cells in insulin production and secretion as described in the Introduction. Unlike primary islets or pancreatic β cells, ERoSHK6 cells are more amenable to long-term culture without compromising their β cell-like functions and are more suitable for assessing the effects of chronic high glucose exposure. As demonstrated by our study, these cells also exhibited a typical β cell dysfunction when exposed to chronic high glucose, i.e. impairment of GSIS [10], [11], [12], [13], [14], [57], [58]. The impairment of GSIS by chronic hyperglycemia is well documented [reviewed by [59]] in patients [60], animals [61], [62], isolated islets [63], [64] and β cell lines [14], [65], [66]. After exposure to chronic high glucose, ERoSHK_HG_ exhibited a substantial increase in basal secretion and concomitant reduction in GSIS consistent with the observations of a previous study using rat islets treated with high glucose for 1 week [64]. In addition, unlike others who reported a 20 to 50% apoptosis in pancreatic β cells within 4–6 days of exposure to high glucose [67], [68], [69], we observed a low but significant increase in apoptosis from 8.01 to 10.94% only after 6 days of treatment. This slower and smaller increase in apoptosis is more consistent with the slow progressive loss in β cell function and β cell mass from apoptosis during T2D pathogenesis.

Comparative analysis of the genome-wide gene expression profile of ERoSHK_LG_ and ERoSHK_HG_ cells revealed that ERoSHK_HG_ cells increased expression of genes predominantly involved in modulating cellular functions such as cellular assembly and organization, cellular growth and proliferation, cell death, cell morphology, and cell-to-cell signaling and interaction. The inclusion of cellular growth and proliferation, and cell death as two of the five most significantly modulated processes by glucose-induced genes was consistent with the well documented effects of glucotoxicity and validated our use of global gene expression to identify candidate cellular functions that are triggered by high glucose. Of the remaining three most significantly modulated processes, it is notable that the common denominator is the involvement of cytoskeleton-mediated structure and this was consistent with the structural abnormalities that have been observed in isolated islets from T2D cadaveric donors [70].

Although the effects of glucose on the modulation of cytoskeleton during insulin secretion have been extensively studied, they have been largely limited to transient glucose stimulation. Little is known about the effects of chronic high glucose on the cytoskeleton of insulin-producing cells.

In this study, we observed that morphological changes in cell shape and colony phenotype were early definitive manifestations of high glucose exposure. Cells within each colony lost their tightly packed arrangement and separated from each other with distinct gaps between adjacent cells in the colony. These changes in colony and cell morphology were underpinned by changes in the actin cytoskeleton, focal adhesion and cell-cell interaction through E-cadherin. Unlike ERoSHK_LG_ colonies where adjacent cells within each colony shared common boundaries demarcated by E-cadherin, many cells in ERoSHK_HG_ colonies were physically separated from each other by distinctly visible gaps between plasma membranes. The gaps between E-cadherin demarcated plasma membranes suggested that high glucose caused disengagement of homotypic E-cadherin interactions. E-cadherin is a cell adhesion molecule and homotypic E-cadherin interactions are important in the aggregation of β cells during islet development in mice [71] or MIN6 pseudoislets. Our observations of disrupted E-cadherin interaction between neighbouring ERoSHK_HG_ cells also implicated for the first time the role of hyperglycemia in disrupting gap junctional communication not only in T2D vulnerable cell types such as vascular smooth muscle, endothelial cells, retinal pericytes and astrocytes [72], [73], [74], [75], [76], [77], [78] but also in pancreatic islets. The importance of E-cadherin in maintaining junctional communication for β cell function has been previously demonstrated by a reduction in GSIS upon down-regulation of E-cadherin in confluent MIN6B1 cell monolayer [79]. Maintaining a tight cell-cell contact of β cells in the islets of Langerhans is also important in forming a syncytium to facilitate the propagation and synchronization of the stimulus-secretion response through the islet via intercellular electrical coupling of membrane depolarization and movement of small signal molecules and ions through tight gap junctions [80], [81] that is crucial in mounting a prompt and appropriate first-phase insulin response to nutrient stimuli [82], [83]. This detrimental effect of hyperglycemia on β cell connectivity is consistent with the well documented “diabetic isletopathy” phenotype in hyperglycemic T2DM patients or rodent models of T2DM [84], [85], [86]. One of the earliest ultrastructural changes in diabetic isletopathy is the loss of adherens junctions and desmosomes, and this loss precedes remodeling fibrosis and islet amyloid deposition [87]. Therefore, the reduced E-cadherin interaction leading to loss of tight cell-cell junction in ERoSHK_HG_ cells represented a mechanism by which GSIS in diabetic patients could be compromised by hyperglycemia.

Staining with fluorescence-tagged phalloidin and antibody against vinculin revealed that unlike ERoSHK_LG_ cells which had basement F-actin stress fibers anchored at vinculin-stained focal adhesion sites, ERoSHK_HG_ cells displayed a disorganized actin cytoskeleton that was no longer anchored at focal adhesion sites. Cortical actin organization in both ERoSHK_LG_ and ERoSHK_HG_ cells was, however morphologically similar. As dispersed pancreatic β-cells which exhibit similar alterations in actin cytoskeleton were reported to have constitutive ERK phosphorylation [79], we investigated ERK1/2 phosphorylation which is downstream of MAPK signaling and has been associated with actin cytoskeleton remodeling, focal adhesion remodeling and GSIS [31], [44]. ERoSHK_HG_ cells were found to have constitutively phosphorylated ERK1/2 and this phosphorylation was refractory to serum deprivation. Inhibition of ERK1/2 phosphorylation by PD98059 reverted the altered actin cytoskeleton, focal adhesions and cell-cell contact in ERoSHK_HG_ cells to that of ERoSHK_LG_ cells. These observations suggested that while HG altered gene expression to support cytoskeletal modification, HG-induced ERK1/2 phosphorylation was a more proximate regulator of the cytoskeleton. The reversal of glucose-induced cytoskeletal abnormalities by PD98059 however did not reverse glucose-induced cell death and impairment of GSIS in ERoSHK_HG_. Instead, cell death and impaired GSIS worsened in both ERoSHK_HG_ and ERoSHK_LG_ cells. This PD98059-mediated cell death and GSIS impairment were consistent with previous reports of its role in inhibiting CREB phosphorylation and initiation of apoptosis [88] and in mediating GLP1-potentiation of GSIS [89]. This perplexing failure of PD98059 to reverse either cell death or impaired GSIS in ERoSHK_HG_ cells could be attributed to the involvement of ERK1/2 phosphorylation in diametrically opposing cellular functions. For example, glucose-induced ERK1/2 phosphorylation has been implicated in the long term deleterious effects of high glucose on β cell, namely apoptosis and impaired insulin secretory function [45] while adiponectin- or insulin-induced ERK1/2 phosphorylation improves cell survival and insulin secretory function [46], [47]. Therefore, ERK1/2 phosphorylation in β cell could be context-dependent, such that phosphorylation in one context leads to regulation of the cytoskeleton while in another context leads to apoptosis and/or GSIS. As such, a global inhibition of ERK1/2 phosphorylation by PD98059 would obscure any context-dependent phosphorylation. One strategy to assess this hypothesis may be to identify the different proteins in the ERK1/2 interactome and determine if ERK1/2-mediated regulation of the cytoskeleton operates through a different signaling cascade of proteins from that of apoptosis or GSIS. If so, it will also support a corollary of this hypothesis that HG-mediated cytoskeletal regulation is decoupled from HG-mediated apoptosis and/or GSIS. However, this would contradict the well-established links between cytoskeleton and GSIS or cytoskeleton and apoptosis.

The critical role of cytoskeleton in stimulated insulin secretion by pancreatic β cells was recognized in the early 1970s [23], [24]. Like all secretory cells, the physical execution of GSIS in pancreatic β cells involves dynamic modulation of the cytoskeleton to move secretory vesicles to the inner surface of the plasma membrane for docking and exocytotic membrane fusion and fission [29]. As such, the cytoskeleton is highly regulated and many components of the cytoskeleton such as focal adhesions [35], F-actin [24], [36], [37], [38], [39], [40], and regulatory small G proteins e.g. Cdc42 and Rac1 [41], [42], [43] have been implicated in the regulation of insulin secretion.

To reconcile our hypothesis that HG-mediated cytoskeletal regulation is decoupled from HG-mediated apoptosis and/or GSIS with the well-established links between cytoskeleton and GSIS or cytoskeleton and apoptosis, we note that stimulated secretion in many secretory cell types correlates with transient depolymerisation of the actin network [30], [40], [44], [90], [91]. Transient remodeling of F-actin cytoskeleton helps mobilize granules to be docked at the plasma membranes [92] and facilitate access of docked insulin granules to the plasma membrane [41], [44]. It has been observed that F-actin undergoes both depolymerization and polymerization during GSIS. For example, F-actin depolymerisation has been shown to enhance GSIS in MIN6 β cells [31] while inhibition of RhoGDI which activate Cdc42 to promote actin polymerization led to increased GSIS [32], [33]. These observations suggest that the F-actin cytoskeleton alternates between polymerization and depolymerization to move, dock and fuse secretory vesicles for GSIS, and a predilection for either a polymerized or depolymerized state would compromise this alternation. Incidentally, an alternating actin polymerization and depolymerization has also been shown to be important in cellular survival [reviewed in [34]]. This requisite for a dynamic cytoskeleton alternating between polymerized and depolymerized provides a rationale for our observations that GSIS and cell survival remained compromised in both ERoSHK_HG_ with a depolymerized F-actin cytoskeleton and in PD98059-treated ERoSHK_HG_ with a polymerized F-actin cytoskeleton.

In conclusion, our approach of using an unbiased global gene expression analysis to uncover candidate β cell functions affected by chronic high glucose exposure re-affirmed the vulnerability of insulin-secreting cells to glucotoxicity and implicated the role of the cytoskeleton in β cell survival and stimulated insulin secretion. Our study also suggested a unifying model linking the cytoskeleton with stimulated insulin secretion and β cell survival. We propose that HG, through modulation of gene expression and ERK1/2 signal transduction, predisposed the actin cytoskeleton in β cells towards a constitutively depolymerized state and compromised the dynamic equilibrium between polymerization and depolymerization of the actin cytoskeleton, resulting in defective stimulated insulin secretion and decreased β cell survival. While inhibition of ERK1/2 signal transduction by PD98059 reversed the actin cytoskeleton towards a constitutively polymerized state, it cannot restore the dynamic equilibrium between polymerization and depolymerization of the actin cytoskeleton, and therefore cannot reverse the compromised insulin secretion and decreased β cell survival.

## Materials and Methods

### Cell culture

Insulin-producing ERoSHK6 cells were derived from mouse embryonic stem cells and cultured as described previously [21]. To investigate the effects of chronic HG exposure as opposed to low glucose (LG) exposure, ERoSHK6 cells were first pre-incubated in INS medium containing 11.1 mM glucose for 24 h, followed by a medium change to one containing either LG (defined as 11.1 mM glucose) or HG (defined as 33.3 mM glucose), and the medium was refreshed every 2 days.

### Insulin secretion assay

Cells were seeded in 12-well plates at 1×10^5^ cells per well and treated with LG or HG for 6 days. They were then washed with Krebs-Ringer bicarbonate buffer with HEPES and 0.1% BSA (KRBH) [43] and pre-incubated in KRBH containing 2.8 mM glucose for 1 h at 37 C. Cells were then incubated in fresh KRBH containing 2.8 mM glucose for 1 h at 37 C (basal secretion), followed by KRBH containing 16.7 mM glucose for 1 h at 37 C (stimulated secretion). The incubation buffers were removed and insulin was extracted from the attached cells using acid-ethanol. Insulin in the incubation buffers and cell extracts was quantified using the rat/mouse insulin ELISA kit (Millipore, Billerica, MA, USA) according to the manufacturer's protocol. Insulin secretion was expressed as a percentage of the total cellular insulin content, which was the sum of insulin present in the basal and stimulated secretions as well as in cell extracts.

### Oligonucleotide microarray

Cells were treated with LG or HG for 6 days. Total RNA from the samples was extracted using the Nucleospin RNA/protein kit (Macherey-Nagel, Düren, Germany). 500 ng of total RNA from each of the biological triplicates of LG or HG-treated cells was reverse transcribed into cDNA, amplified to generate biotinylated cRNA and purified using the Illumina TotalPrep RNA amplification kit (Ambion Inc., Austin, TX, USA) according to the manufacturer's protocol. Hybridization to the Illumina MouseRef-8 v2.0 Expression BeadChip (Illumina Inc., San Diego, CA, USA), washing and scanning were performed according to the Illumina BeadStation 500× manual. Data were extracted and average normalization and background subtraction was performed using Illumina BeadStudio Gene Expression Module v3 provided by the manufacturer. Transcript signals below the limit of detection of 95% confidence were defined as genes unexpressed, and an expression fold change criteria of 2.0 was used to determine up- or down-regulation of genes. Gene datasets were uploaded onto Ingenuity Pathway Analysis platform (Ingenuity Systems, Mountain View, CA, USA) for functional clustering. Gene expression changes were validated by analyzing expression levels of randomly selected genes using quantitative PCR (qPCR). Briefly, 1 µg of RNA was converted to cDNA using random primers in a 20-µl reaction volume using a High Capacity cDNA Archive Kit (Applied Biosystems, Foster City, CA, USA). A 20 µl qPCR reaction mixture was set up to contain 20 ng of cDNA in a 3-µl volume, 250 nM of both forward and reverse primers in a 2-µl volume, 5 µl of nuclease-free water and 10 µl of 2× SYBR Green PCR Master Mix (Applied Biosystems). Amplification was performed on ABI StepOne Plus Real Time PCR System (Applied Biosystems). Relative transcript levels were calculated based on comparative changes in cycle threshold values, with *Actb* as the endogenous control. Primer sequences are listed in Table S1.

### Total viable cell count

Cells were seeded in 24-well plates at 2×10^5^ cells per well and treated with LG or HG. At each indicated timepoint, triplicate wells in each treatment group were trypsinized into single-cells and total viable cell number was counted using the Guava ViaCount assay on a Guava Personal Cell Analysis-96 system (Guava Technologies, Hayward, CA, USA).

### Cell cycle analysis

Cells were treated with LG or HG for 6 days and cell cycle analyses were performed at the indicated timepoints for a period of 72 h thereafter. At each timepoint, they were harvested by trypsinization into single-cells. 5×10^5^ cells were washed and fixed in 70% ethanol at −20 C overnight. They were then collected by centrifugation, resuspended in PBS containing 5 µg/ml RNase, DNase-free (Roche Applied Science Inc., Penzberg, Upper Bavaria, Germany) and 0.05% Triton X-100 and incubated for 2 h at 37 C with rotation. Subsequently, propidium iodide was added to a final concentration of 50 µg/ml and DNA content of the cells was measured using the BD FACSCalibur flow cytometer (BD Biosciences, Franklin Lakes, NJ, USA). Histograms were deconvoluted using the ModFit LT v3.2 software (Verity Software House, Topsham, ME, USA).

### Cell division assay

Cells were subjected to LG or HG treatment for 6 days, trypsinized into single-cells and incubated with 5 µM of carboxyfluorescein diacetate, succinimidyl ester (CFSE, Invitrogen) for 15 min at 37 C. They were washed to remove excess dye, resuspended in INS medium containing 2 or 6 g/l glucose and plated at a density of 1×10^6^ cells per well in 6-well plates. Cells were then harvested at the indicated timepoints (0–72 h post CFSE-staining), fixed in 2% paraformaldehyde and the median fluorescence intensity of the cells was measured using the BD FACSCalibur flow cytometer (BD Biosciences). The number of cell divisions (*n*) at each timepoint was calculated as described in [52]. Briefly, *n* = (lg F_0_/F_n_)/lg 2 where F_0_ is the initial average cellular fluorescence and F_n_ is the average cellular fluorescence at each timepoint. When time (h) was plotted against *n*, the gradient of the linear graph represents the duration of one cell division (h).

### Annexin V assay

Cells were subjected to LG or HG treatment for 6 days and Annexin V assays were performed at the indicated timepoints for a period of 72 h thereafter. At each timepoint, they were harvested by trypsinization into single-cells. 2×10^5^ cells were washed and stained using the Annexin V-PE Apoptosis Detection Kit (Merck, KGaA, Darmstadt, Germany) according to the manufacturer's protocol. The percentage of Annexin V-positive cells was quantified on the BD FACSCalibur flow cytometer (BD Biosciences).

### Immunocytochemistry

Cells were seeded onto 20×20 mm glass coverslips, subjected to LG or HG treatment for 6 days, fixed with 4% paraformaldehyde for 15 min and then permeabilized with 0.1% Triton X-100 for 3 min. The cells were then incubated with 4% normal goat serum for 30 min to block non-specific binding sites, and then incubated with 1∶100 diluted purified mouse anti-vinculin monoclonal IgG_1_ clone 7F9 (Millipore) or purified mouse anti-E-cadherin monoclonal IgG_2a_ clone 36 (BD Biosciences) at 4 C overnight. Cells were subsequently washed and incubated with 1∶500 diluted Alexa Fluor 488-conjugated goat anti-mouse IgG antibodies (Invitrogen, Carlsbad, CA, USA) and 1∶500 diluted rhodamine-conjugated phalloidin (Millipore) for 1 h. They were then washed, counterstained with DAPI, mounted and visualized using a Zeiss LSM 510 laser scanning confocal microscope (Carl Zeiss, Inc., Oberkochen, Germany).

### ERK phosphorylation assay

Serum starvation was performed by incubating cells in KRBH containing 2.8 mM glucose. Cultured cells were washed with PBS twice and proteins were extracted using the Mammalian Cell Extraction Kit (BioVision, Mountain View, CA, USA) supplemented with Phosphatase Inhibitor Cocktail 3 (Sigma-Aldrich, St. Louis, MO, USA) according to the manufacturer's protocol. Total protein amount was measured using the Bio-Rad Protein Assay (Bio-Rad, Hercules, CA, USA). The proteins were electroblotted onto a nitrocellulose membrane after first separating on a 4–12% SDS–polyacrylamide gel. The membrane was blocked with StartingBlock T20 (PBS) Blocking Buffer (Thermo Fisher Scientific, Rockford, IL, USA) for 30 min at room temperature and incubated with 1∶1000 diluted purified rabbit anti-phospho-ERK1/2 (Thr202/Tyr204) polyclonal IgG (Cell Signaling Technology, Danvers, MA, USA). The blot was then washed and incubated with a horseradish peroxidase-conjugated 1∶5000 diluted goat anti-rabbit IgG (Santa Cruz Biotechnology, Santa Cruz, CA, USA). The blot was then washed and incubated with HRP-enhanced chemiluminescent substrate (Thermo Fisher Scientific) and exposed to an X-ray film. To probe for total ERK1/2, bound antibodies were stripped off the blot by incubating it in Restore Western Blot Stripping Buffer (Thermo Fisher Scientific) for 5 min at room temperature. A second western blot was performed using 1∶1000 diluted purified rabbit anti-ERK1/2 polyclonal IgG clone K-23 (Santa Cruz Biotechnology) as primary antibodies instead. Intensities of protein bands were quantified by densitometry using ImageJ version 1.44d (NIH, USA).

### Statistical analyses

Statistical significance between two samples was determined using unpaired Student's *t* tests, with *P*<0.05 as the level of significance.

## Supporting Information

Figure S1 **Cell division rate of ERoSHK_LG_ and ERoSHK_HG_.** ERoSHK cells pretreated with LG and HG for 6 days were stained with CFSE and the rate of loss of fluorescence signal was monitored over 72 h by flow cytometry. Duration of cell division can be calculated based on the gradient of the best-fit line of the *time* versus *n* scatter plot, where *n* is the number of cell divisions at each timepoint (see Materials and Methods).
(TIF)

Figure S2 **Inhibition of ERK1/2 signaling did not reduce apoptosis in ERoSHK_HG_.** ERoSHK cells were treated with LG and HG in combination with various concentrations of the ERK1/2 inhibitor PD98059 for 6 days. The cells were then stained with phycoerythrin-conjugated Annexin V and dye fluorescence was measured by flow cytometry. Percentages of Annexin V-positive cells are assessed and graphically represented. Data are presented as mean ± s.d.; n = 3.
(TIF)

Table S1 **Primer sequences for qPCR.**
(DOCX)

Table S2 **List of genes upregulated by >2.0 fold in ERoSHK_HG_**
(XLS)

Table S3 **Genes clustered into each of the top 5 most significant cellular processes in ERoSHK_HG_**
(XLSX)
