# Supplementary figures and images for: High Glucose Predisposes Gene Expression and ERK Phosphorylation to Apoptosis and Impaired Glucose-Stimulated Insulin Secretion via the Cytoskeleton
Source: PLoS One. 2012 Sep 14;7(9):e44988. doi: 10.1371/journal.pone.0044988 (PMC3443235; doi:10.1371/journal.pone.0044988)

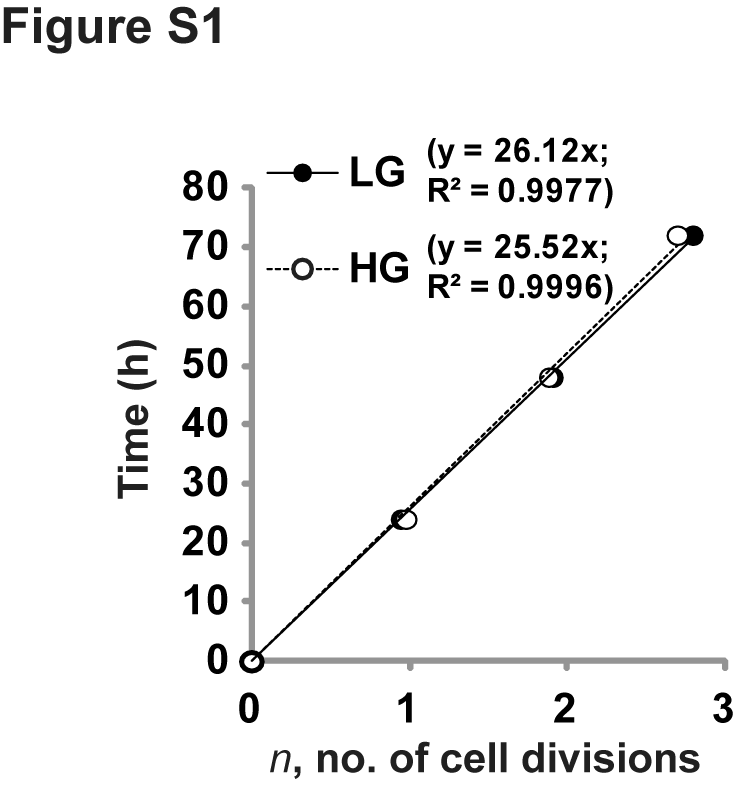

Supplement: Figure S1 — Cell division rate of ERoSHKLG and ERoSHKHG. ERoSHK cells pretreated with LG and HG for 6 days were stained with CFSE and the rate of loss of fluorescence signal was monitored over 72 h by flow cytometry. Duration of cell division can be calculated based on the gradient of the best-fit line of the time versus n scatter plot, where n is the number of cell divisions at each timepoint (see Materials and Methods). (TIF) [file pone.0044988.s001.tif]

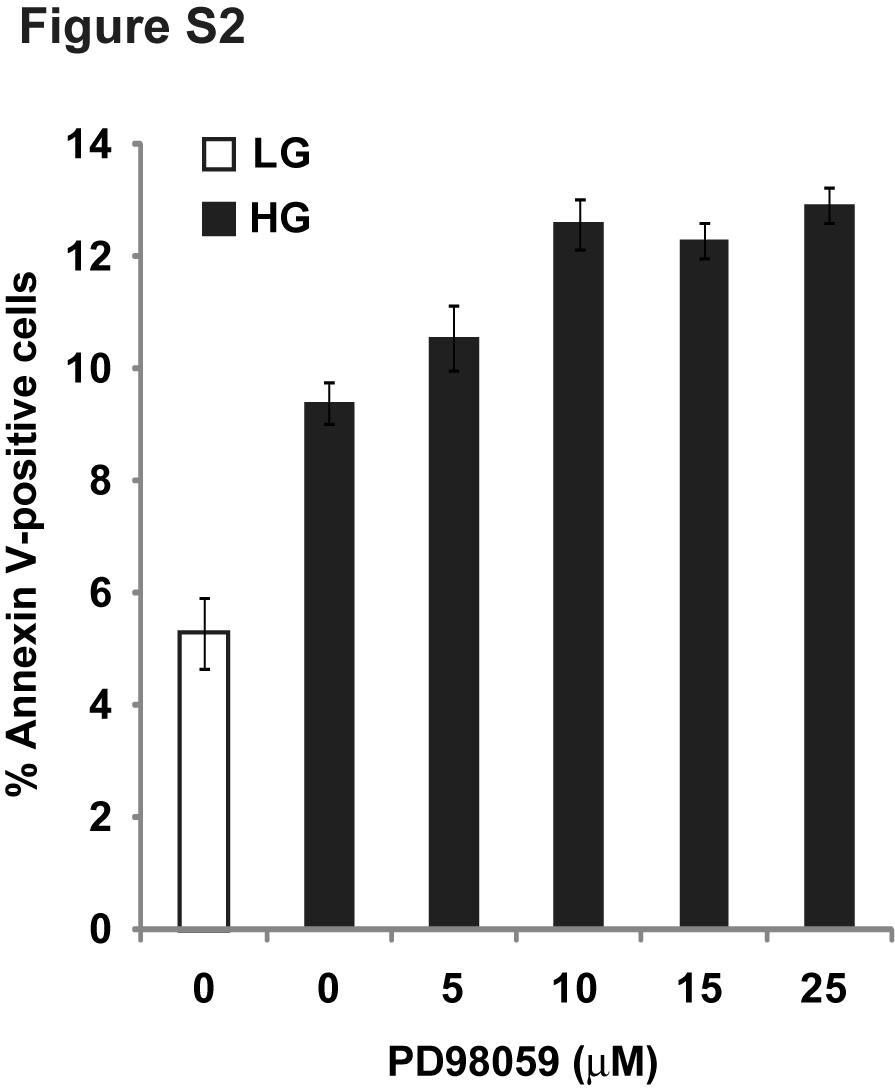

Supplement: Figure S2 — Inhibition of ERK1/2 signaling did not reduce apoptosis in ERoSHKHG. ERoSHK cells were treated with LG and HG in combination with various concentrations of the ERK1/2 inhibitor PD98059 for 6 days. The cells were then stained with phycoerythrin-conjugated Annexin V and dye fluorescence was measured by flow cytometry. Percentages of Annexin V-positive cells are assessed and graphically represented. Data are presented as mean ± s.d.; n = 3. (TIF) [file pone.0044988.s002.tif]
