# Supplementary material for: High Glucose Predisposes Gene Expression and ERK Phosphorylation to Apoptosis and Impaired Glucose-Stimulated Insulin Secretion via the Cytoskeleton
Source: PLoS One. 2012 Sep 14;7(9):e44988. doi: 10.1371/journal.pone.0044988 (PMC3443235; doi:10.1371/journal.pone.0044988)
Supplement: Table S1 — Primer sequences for qPCR. (DOCX) [file pone.0044988.s003.docx]

**Table S1. Primer sequences for qPCR.**

| Primer | Sequence (5’ to 3’) |
| --- | --- |
| Actn2-158-fp | TGAATCAGATAGAGCCCGGCGTG |
| Actn2-158-rp | CCGCAGGTGTGAGTTGCACC |
| Tpm2-158-fp | TACCAACAACTTGAAATCCCTGG |
| Tpm2-158-rp | TGGTTTTCTCCAACTTTGCCAC |
| Cdkn1a-126-fp | GTGGCCTTGTCGCTGTCTT |
| Cdkn1a-126-rp | GCGCTTGGAGTGATAGAAATCTG |
| Wnt5a-115-fp | AGCCTGTAAGTGTCATGGAGT |
| Wnt5a-115-rp | CGCGGCGCTATCATACTTCT |
| Dapk2-145-fp | CAGCCTTTCGCCGATTGTATGGTC |
| Dapk2-145-rp | TCTCCCGGCACTTCTTCACGATG |
| Ppargc1a-188-fp | CGTAGGCCCAGGTACGACAGCT |
| Ppargc1a-188-rp | TCTGTCCGCGTTGTGTCAGGT |
| Mylip-158-fp | ACCTTACCGCCTTAAACTGAGG |
| Mylip-158-rp | GGAGGGCACTGAGTTCCAC |
| Ninj2-137-fp | AACCATCCACCTTCAACACAGGCA |
| Ninj2-137-rp | TCAGCCGCATGGCATTGGACA |
| Cd97-101-fp | CTCCCCGAGCAGACAACTAC |
| Cd97-101-rp | CAATGGTTTTGCCCGGAGAT |
| Ephb3-135-fp | CATGAATCCTATCCGCACGTATC |
| Ephb3-135-rp | CAGTCTCTTACGGTGAACTTCAG |
